# Efficacy of Exercise Rehabilitation Program in Relieving Oxaliplatin Induced Peripheral Neurotoxicity

**DOI:** 10.31557/APJCP.2021.22.3.705

**Published:** 2021-03

**Authors:** Qi Gui, Dapeng Li, Yan Zhuge, Chengcheng Xu

**Affiliations:** 1 *Departments of Oncology, The First Affiliated Hospital of Soochow University, Suzhou, 215006, Jiangsu, People’s Republic of China. *; 2 *Departments of Thoracic Surgery, The First Affiliated Hospital of Soochow University, Suzhou, 215006, Jiangsu, People’s Republic of China. *

**Keywords:** Oxaliplatin, peripheral neurotoxicity, exercise, rehabilitation

## Abstract

**Background:**

Peripheral neurotoxicity is common in patients with digestive malignancies receiving chemotherapy containing oxaliplatin, and there is still no effective drug to prevent or treat this complication.

**Methods:**

Seventy-nine patients receiving chemotherapy containing oxaliplatin were included, and the relationship between chemotherapy regimens, cycles, and cumulative dose of oxaliplatin and peripheral neurotoxicity was analyzed. Patients were divided into two groups of control or intervention. Twenty-eight patients in the control group received routine chemotherapy care, and 51 patients in the intervention group underwent two-week exercise rehabilitation program. Patients’ Functional Assessment of Cancer Therapy/Gynecologic Oncology Group – Neurotoxicity (FACT/GOG-Ntx), functional tests, and Brief Pain Inventory(BPI) scores as well as interference life scores were assessed before intervention and two weeks after the intervention.

**Results:**

In the intervention group, 52.9% patients previously exercised regularly. The FOLFOX regimen was more common in peripheral neurotoxicity (73.4%), and the median oxaliplatin cycles for neurotoxicity was 9 (ranging from 1 to 16). The mean cumulative dose of oxaliplatin was 1080.02 ± 185.22 mg, both the cycles and cumulative dose were positively correlated with the occurrence of peripheral neurotoxicity. Compared with control, the scores of FACT/GOG-Ntx, functional tests, and BPI were significantly decreased in the intervention group (p < 0.05).

**Conclusion:**

Chemotherapy cycles and cumulative doses were in relation with OIN , and exercise rehabilitation program could effectively alleviate OIN.

## Introduction

Oxaliplatin is a platinum analogue, which forms a cross-link with DNA to antagonize its replication and transcription (Woynarowski et al., 2000). Currently, oxaliplatin is widely used in the treatment of malignant tumors in digestive system, such as colorectal, gastric, and pancreatic cancers (Nagata et al., 2019). The canonical regimens containing oxaliplatin were in combination of 5-fluorouracil (5-FU) and tetrahydrofolate into the FOLFOX regimen and in combination with oral capecitabine into the XELOX regimen (Chang and Abbruzzese, 2005). Although patients well tolerate common adverse effects after oxaliplatin-based chemotherapy, such as myelosuppression, nausea, and vomiting, they always suffer from the main adverse effect of oxaliplatin, peripheral neuropathy, which is mainly characterized by distal limb paresthesia, numbness, and sock- and glove-like distribution (Besora et al., 2018). It can be manifested as sensory ataxia and dysfunction (Andriamamonjy et al., 2017), and may lead to insufficient dosages of chemotherapy or even cessation of treatment, severely decreasing patients’ quality of life and efficacy of treatment (Raphael et al., 2017). There are few clinical studies on the relationship between dose and cycles of chemotherapy and peripheral neurotoxicity, and no effective drug to prevent or treat OIN has been introduced yet (Stefansson and Nygren, 2016). This prospective study analyzed the clinical and behavioral features of OIN and evaluated the efficacy of exercise rehabilitation care in alleviating peripheral neurotoxicity induced by oxaliplatin .

## Materials and Methods

*Patients*

**Figure 1:**
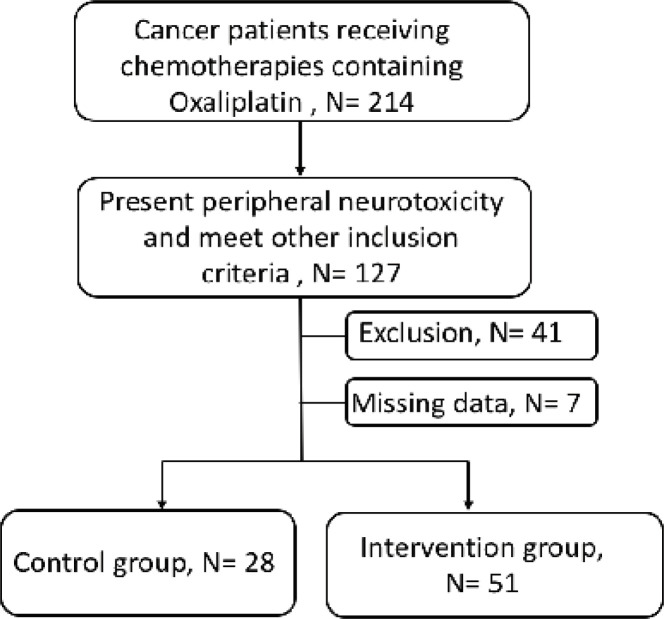
Patient Profile

Patients received oxaliplatin-based chemotherapy at the Department of Oncology in the First Affiliated Hospital of Soochow University were enrolled in this study. Other main inclusion criteria were presenting different degrees of peripheral neurotoxicity during or after chemotherapy, aging from 18 to 60 years old, having pathologically confirmed malignant tumor, and willingness to participate in the study. Exclusion criteria were having history of neurological diseases or diabetes, receiving other treatments that might cause peripheral neurotoxicity, combined with vertebral metastases and/or intracranial metastases, and having merging cognitive impairment or mental disorder. Patients could choose to receive routine care or 2 weeks of exercise rehabilitation program on the basis of routine care. Patients’ clinical and behavior characteristics were retrospectively reviewed from their medical record. From July 2018 to December 2018, 214 cancer patients receiving chemotherapies of oxalipatin were enrolled. Among them, 127 patients presented peripheral neurotoxicity and met our inclusion criteria. With respect to our exclusion criteria, 41 patients were excluded. The remain 86 patients underwent further investigation. Seven patients were also excluded due to incomplete data. Finally, 79 patients were divided into two groups of control (n=28) and intervention (n=51) based on patients’ choices (Figure 1). 

*Patient groups*

The patients in the control group received regular care: patients with oxaliplatin infusion should keep warm, do not drink cold water, do not touch cold objects, and use central venous catheters to avoid local extravasation of chemotherapy drugs (Sorich et al., 2004).

Patients in intervention group received 2 weeks of exercise rehabilitation program on the basis of regular care. In brief, the exercise rehabilitation program is a comprehensive gymnastics and quickly walking training. The program in details was as follows: a. doing comprehensive gymnastics training 3 times every morning and evening (lying down, moving hands and fingers and feet toes in turn 10 times, then stand, slowly raising arms and then stretching out and drawing back fingers 10 times, and then slowly falling arms; at last, placing hands on hips, heeling slowly after falling off the ground 10 times) and b. quick-step walking training(according to the patient’s own physical condition, patients could choose to walk quickly from 1 to 3 km). During exercise, if patients felt dizziness, chest tightness, or other physical discomfort, exercising was immediately stop.

*Peripheral neurotoxicity assessment*

The 4^th^ edition of the FACT/GOG-Ntx scale was used to assess the subjective symptoms of neuropathy (McCrary et al., 2017). The FACT/GOG-Ntx scale has a total of 11 items, including sensory, auditory, motor, and dysfunction. The patient scores from 0 to 4 according to his/her degree of symptoms: 0 (not at all), 1 (a little), 2 (occasionally), 3 (often), and 4 (very frequently). The total score was calculated according to the principles in the FACT (Functional Assessment of Cancer Therapy) manual.

*Functional assessment*

In order to test the effect of neurotoxicity on the patients’ physical function, we calculatedthe time of the 6-hole shirt (the patient puts on a 6-hole shirt, and twist 6 buttons as soon as possible), the fastest time to do fifty steps walking, and the time of the coin test (the patient sat at the table, picked up 4 coins in turn, and put them in the cup one by one) (Griffith et al., 2017).

*Brief Pain Inventory (BPI)*

 BPI is a questionnaire that effectively assesses patients’ subjective pain symptoms. The first 2 questions are to assess the severity of pain 0 (no pain) and 10 (the most imaginable pain). For the third question (using the interference scale), patients are required to score their previous 24-hour life interference because of pain using score 0 (no interference at all) to 10 (all interfered) (El-Fatatry et al., 2018).

*Statistical analysis*

In this study, chi-square test was used to compare the general characteristics of the two groups. Covariance analysis was also used to compare the FACT/GOG-Ntx scores, functional test scores, and BPI scores between the two groups. Moreover, chi-square test was used to compare the chemotherapy regimens between control group and intervention group. The t-test was also used to compare the cycles and the cumulative dose of oxaliplatin. According to SPSS (version 19.0) , p < 0.05 was considered as a statistically significant difference.

## Results

*The clinical characteristics of two groups*

 Intriguingly, 32 patients previously exercised regularly, and most of them (n=27, 84.3%) were in the intervention group. In control group, there were 18 males and 10 females, and the patients’ mean age was 52 years old (ranging from 41 to 60). In intervention group, there were 34 males and 17 females, and the patients’ mean age was 50 years old (ranging from 39 to 60). There was no statistically significant difference between two groups regarding age, gender, history of surgery, history of radiotherapy, and tumor type (p > 0.05) (Table 1). 

*The relationship between cycles and cumulative dose and OIN*

Among chemotherapy regimens presenting peripheral neurotoxicity, the most ones were FOLFOX regimens (including FOLFOX4, FOLFOX6, mFOLFOX6) (73.4%), followed with XELOX (12.7%), FOLFIRINOX (7.6%), and EOX (6.3%) regimen. There were no significant differences between the two groups in the cases of chemotherapy regimens, cycles, and doses (p > 0.05). The median cycle of OIN emergence was 9 (ranging from 1 to 16), and the cumulative dose of oxaliplatin was 1080.02 ± 185.22 mg. The cycles and cumulative dose were positively correlated with the occurrence of peripheral neurotoxicity (Table 2). 

*Exercise rehabilitation and neurological symptoms, functional assessments, and pain scores of OIN*

After a two-week exercise rehabilitation program in intervention group, the patients’ FACT/GOG-Ntx, functional test, and BPI scores were significantly decreased (p < 0.05). In addition, exercise rehabilitation program significantly improved patients’ walking abilities, normal work abilities , and sleep quality (p < 0.05). However, patients’ general activities, mood, relationship with others, and life enjoyment did not change significantly (p > 0.05). In control group, the FACT/GOG-Ntx, functional tests, and BPI scores as well as pain disturbances did not improve compare to baseline (p > 0.05) (Table 3).

**Table. 1:** Baseline Characteristics of the Two Groups of Patients

	Control (n=28)	Intervention (n=51)
Mean age ± SD	52 ± 7	50 ± 8
Male n (%)	18 (64.3)	34 (66.7)
History of surgery (yes) n (%)	20 (71.4)	39 (76.5)
History of radiotherapy (yes)n (%)	6 (21.4)	12 (23.5)
Regular exercise behavior (yes), n (%)	5 (17.9)	27 (52.9)
Tumor types n (%)		
Colorectal cancer	17 (60.7)	32 (62.7)
Gastric cancer	7 (25.0)	11 (21.5)
Pancreatic cancer	2 (7.1)	5 (9.8)
Liver cancer	2 (7.1)	3 (5.9)

**Table. 2:** The Clinical Analysis of Oxaliplatin Induced Peripheral Neurotoxicity

	Control (n=28)	Intervention (n=51)	Total (n=79)
Chemotherapy regimens n (%)			
FOLFOX regimens	20 (71.4)	38 (74.5)	58 (73.4)
XELOX	4 (14.3)	6 (11.7)	10 (12.7)
FOLFIRINOX	2 (7.1)	4 (7.8)	6 (7.6)
EOX	2 (7.1)	3 (5.9)	5(6.3)
Median cycles of OIN appearance (range)	8 (2-15)	9 (1-16)	9 (1-16)
Cumulative dose of Oxaliplatin (mg, mean (SD))	1082.22 ± 167.15	1076.18 ± 198.02	1080.02 ± 185.22

**Table 3:** Neurological Symptoms, Functional Assessment, and Pain Scores at Baseline and after 2 Weeks in Both Groups

	Control		Intervention	
	Baseline	After two-weeks	Baseline	After two-weeks
FACT/GOG-Ntx	16.9 ± 4.1	17.4 ± 3.7	17.1 ± 4.4	15.0 ± 3.6*
Functional assessment				
6-Hole Shirt Time	40.1 ± 4.0	39.5 ± 4.9	41.2 ± 4.6	35.8 ± 4.7*
Walking 50 Steps	25.1 ± 4.8	24.4 ± 4.4	25.2 ± 4.9	19.9 ± 4.5*
Coin Test	6.5 ± 1.1	6.4 ± 1.1	6.8 ± 1.1	5.9 ± 0.9*
BPI Scores				
Worst Pain	2.2 ± 1.3	2.1 ± 1.3	2.4 ± 1.4	1.7 ± 1.2*
Mean Pain	1.1 ± 0.7	1.2 ± 0.8	1.3 ± 0.9	0.8 ± 0.7*
Pain Disturbances				
General Activities	1.0 ± 0.7	1.2 ± 0.8	1.2 ± 0.9	1.1 ± 0.9
Mood	1.0 ± 0.8	1.2 ± 0.9	1.2 ± 0.9	1.1 ± 0.8
Walking Abilities	1.4 ± 0.9	1.5 ± 1.0	1.4 ± 0.9	0.9 ± 0.7*
Normal Work	2.1 ± 1.2	2.1 ± 1.1	2.2 ± 1.3	1.7 ± 1.3*
Relationship	0.8 ± 0.7	0.8 ± 0.8	0.7 ± 0.6	0.8 ± 0.7
Sleep	1.7 ± 1.2	1.9 ± 1.2	1.8 ± 1.1	1.2 ± 0.9*
Life Enjoyment	2.1 ± 1.4	1.9 ± 1.0	2.0 ± 1.2	2.0 ± 1.3

* indicate that there was a statistically significant difference (p < .05) between the baseline and the 2 weeks after treatment.

## Discussion

Oxaliplatin induced peripheral neurotoxicity is clinically manifested in both acute and chronic forms, with a higher incidence of its acute form ranging from 65% to 100% (El-Fatatry et al., 2018). The typical manifestations of acute neurotoxicity are distal limb or perioral dysfunction and dull sensation of the throat which are induced by cold stimulation (Banach et al., 2018). These syndromes usually occur before oxaliplatin infusion, during the infusion, or soon after the infusion. Most of these patients can recover within hours or days (Velasco et al., 2015). In contrast, chronic neurotoxicity caused by oxaliplatin is a dose-cumulative neuropathy which affects up to 80% of patients. It severely affects patients’ life, reduces patients’ compliance, and may lead to reduced dose and/or early withdrawal of chemotherapy, reducing the efficacy of anti-tumor therapy (Ma et al., 2018).

A meta-analysis was done on 3,869 patients who received oxaliplatin regimens in 14 studies. The results showed that only six studies evaluated the relationship between neurotoxicity and oxaliplatin cumulative dose, and among them, five revealed that neurotoxicity was associated with cumulative doses (Beijers et al., 2014). This study showed that the chemotherapy cycles of presenting peripheral neurotoxicity was 8 to 9 in patients receiving oxaliplatin-based chemotherapy regimens, with a mean cumulative dose of 1,080.02 ± 185.22 mg. The most frequent regimen for chronic peripheral neurotoxicity was FOLFOX. These findings were consistent with a previous study (Palugulla et al., 2017). To reduce peripheral neurotoxicity, the IDEA collaborative study extracted six clinical studies and evaluated the non-inferiority of FOLFOX/CAPOX as adjunctive therapy with a short course of treatment (3 months vs. 6 months). In this study, clinically relevant OIN was significantly reduced in the 3-month group (FOLFOX and CAPOX were 16.6% vs. 47.7%, and 14.2% vs. 44.9%, respectively), but the non-inferior results of shortening the course of treatment were not confirmed in the overall population, and 3-year disease-free progression was reduced by 0.9% (Grothey et al., 2018). 

The mechanism through which oxaliplatin causes peripheral neurotoxicity remains unclear. The possible mechanisms are axonal over-excitation, voltage-gated sodium and/or potassium channel changes leading to repetitive discharge and oxidative stress (Argyriou et al., 2019; Poupon et al., 2018). The effects of dorsal root ganglia caused by accumulated into neuronal damage (Beijers et al., 2014). Currently, there are no standard drugs or methods to effectively treat OIN. Previous studies have shown that exercise can promote the regeneration of peripheral nerves (Streckmann et al., 2019). Furthermore, in a neuropathic mouse model (Park et al., 2015), exercise could prevent the occurrence of peripheral neuropathy and in a mouse model of diabetic peripheral neuropathy, exercise could delay disease progression (Groover et al., 2013). However, no study evaluated the role of exercise on OIN. Therefore, we speculate that exercise may accelerate peripheral blood circulation, which can accelerate metabolism of chemotherapy drugs, reduce toxic drug damage, and promote peripheral nerve to regenerate. Our exercise rehabilitation program is simple and easily to do with the designed movements dominated by fingers and toes. Basic research has found that sensory axonal damage reduces the amplitude of sensory nerve action potentials, while most motor nerve functions are unaffected. Therefore, the motor function of patients with OIN has not been affected, and most rehabilitation exercises can be sustained and completed.

However, there were also limitations with this study. Since more patients who previously exercised regularly were in the intervention group. Future studies using larger sample size is needed to further confirm the efficacy of exercise in relieving OIN, or to discover which types of exercise may be helpful. Nevertheless, we provide some evidence that exercise rehabilitation program can effectively alleviate OIN. 

## Author Contribution Statement

The authors confirm contribution to the paper as follows: study conception and design: C. Xu and Q. Gui; data collection: D. Li and Y. Zhuge; analysis and interpretation of results: Q. Gui and D. Li; draft manuscript preparation: C. Xu, Q. Gui and D. Li. All authors reviewed the results and approved the final version of the manuscript.
